# Post-Viral Pain, Fatigue, and Sleep Disturbance Syndromes: Current Knowledge and Future Directions

**DOI:** 10.1080/24740527.2023.2272999

**Published:** 2024-01-09

**Authors:** Caleb Tackey, P. Maxwell Slepian, Hance Clarke, Nimish Mittal

**Affiliations:** a Faculty of Medicine, University of Toronto, Toronto, Ontario, Canada; b Department of Anesthesiology and Pain Medicine, University of Toronto, Toronto, Ontario, Canada; c Department of Anesthesia and Pain Management, Toronto General Hospital, University Health Network, Toronto, Ontario, Canada; d Division of Physical Medicine and Rehabilitation, Department of Medicine, University of Toronto, Toronto, Ontario, Canada; e Faculty of Kinesiology and Physical Education, University of Toronto, Toronto, Ontario, Canada

**Keywords:** viral, post-viral, pain, fatigue, sleep

## Abstract

**Background:**

Post-viral pain syndrome, also known as post-viral syndrome, is a complex condition characterized by persistent pain, fatigue, musculoskeletal pain, neuropathic pain, neurocognitive difficulties, and sleep disturbances that can occur after an individual has recovered from a viral infection.

**Aims:**

This narrative review provides a summary of the sequelae of post-viral syndromes, viral agents that cause it, and the pathophysiology, treatment, and future considerations for research and targeted therapies.

**Methods:**

Medline, PubMed, and Embase databases were used to search for studies on viruses associated with post-viral syndrome.

**Conclusion:**

Much remains unknown regarding the pathophysiology of post-viral syndromes, and few studies have provided a comprehensive summary of the condition, agents that cause it, and successful treatment modalities. With the COVID-19 pandemic continuing to affect millions of people worldwide, the need for an understanding of the etiology of post-viral illness and how to help individuals cope with the sequalae is paramount.

## RÉSUMÉ

**Contexte:** Le syndrome de la douleur post-virale, également connu sous le nom de syndrome post-viral, est une affection complexe caractérisée par des douleurs persistantes, de la fatigue, des douleurs musculosquelettiques, des douleurs neuropathiques, des difficultés neurocognitives et des troubles du sommeil qui peuvent survenir après la guérison d’une infection virale.

**Objectifs:** Cette revue narrative présente un résumé des séquelles des syndromes post-viraux, des agents viraux qui les causent, ainsi que de la pathophysiologie, des traitements et des considérations futures pour la recherche et les traitements ciblés.

**Méthodes utilisées:** Les bases de données Medline, PubMed et Embase ont été utilisées pour rechercher des études sur les virus associés au syndrome post-viral.

**Conclusion:** La physiopathologie des syndromes post-viraux reste largement méconnue et peu d’études ont présenté un résumé complet de l’affection, des agents qui la provoquent et des modalités de traitement efficaces. Alors que la pandémie de COVID-19 continue d’affecter des millions de personnes dans le monde, il est primordial de comprendre l’étiologie de la maladie post-virale et de savoir comment aider les individus à faire face aux séquelles.

## Introduction

ost-viral pain syndrome, also known as post-viral syndrome (PVS), is a complex condition characterized by persistent pain, fatigue, musculoskeletal pain, neuropathic pain, neurocognitive difficulties, and sleep disturbances^1,2^ that can occur after an individual has recovered from a viral infection. The condition can last for weeks, months, or even years and can significantly impact the individual’s quality of life. Cases of PVS have been documented after influenza B,^3^ Ebola,^4^ Chikungunya,^5^ Dengue,^6^ and many other infections; however, much remains unknown regarding the pathophysiology of PVSs, and few studies have provided a comprehensive summary of the condition, agents that cause it, and successful treatment modalities.

With the COVID-19 pandemic continuing to affect millions of people worldwide, the need for an understanding of the etiology of post-viral illness and how to help individuals cope with the sequalae is paramount.^2^ A significant number of patients who were infected by SARS-CoV-2 continued to experience symptoms long after the acute phase of their disease.^7^ Providing care for patients suffering from a PVS will place a significant burden on already-strained health care systems globally.^2,8^ For example, in Latvia, COVID-19 is predicted to increase the economic burden of chronic fatigue syndrome (CFS) by at least 15%.^9^ To adequately cope with the numbers of patients who will present with post-COVID-19 syndromes, health care practitioners must familiarize themselves with the causes of PVS and how it can be effectively diagnosed and managed. This narrative review will provide a summary of the sequelae of PVSs, viral agents that cause it, and the pathophysiology and treatment.

## Post-Viral Pain

### Viral Agents Associated with Post-Viral Pain

Pain is one of the most common symptoms that can persist after a viral infection has been cleared. The pain can be either musculoskeletal in origin (e.g., myalgia, arthralgia)^5,6,10^ or neuropathic (e.g., myelitis, allodynia, postherpetic neuralgia).^11,12^ In some cases, pain begins during the acute phase of the viral infection and continues into the postinfectious phase.^11^ There are other cases in which new-onset pain develops weeks to months after recovery from infection.^6,13^ The presentation of the pain varies vastly depending on the viral agent that caused the infection (Table 1).

**Table 1.** Summary of viral agents associated with post-viral syndrome.

Virus	Discriminatory signs and symptoms	Diagnostic tools	Treatment for post-viral symptoms
COVID-19	Acute stage: Fever, cough, dyspnea, fatigue, myalgia Post-viral stage: Myalgia, arthralgia, chronic fatigue, neuropathic pain, sleep disorder, depression, anxiety, PTSD	RT-PCR	Aerobic activity CBT BBTI APAP Antidepressants Gabapentinoids Systemic corticosteroids IVIG
CHIKV	Acute stage: Fever, rash, arthralgia Post-viral stage: Arthralgia, myelitis	RT-PCR	Physiotherapy NSAIDs Systemic corticosteroids DMARDs (e.g., methotrexate)
VZV	Acute stage: Pruritic fluid-filled vesicles Post-viral stage: Dermatomal rash, postherpetic neuralgia	PCR	Mindfulness CBT Antivirals Gabapentinoids TCAs Serotonin–norepinephrine reuptake inhibitors Opioids Cannabinoids
Poliovirus	Acute stage: Fatigue, headache, neck stiffness Post-viral stage: Myalgia, arthralgia, neuropathic pain, functional decline, fatigue	RT-PCR	IVIG
HIV	Acute stage: Myalgia, arthralgia, weight loss, cervical lymphadenopathy Post-viral stage: Allodynia, anxiety, depression, PTSD	RT-PCR Nucleic acid–based amplification assay	CBT

RT-PCT = reverse transcription polymerase chain reaction; DMARD = disease-modifying antirheumatic drug; PCR = polymerase chain reaction.

Chikungunya virus (CHIKV) is a single-stranded RNA virus that is mosquito-transmitted and was first isolated in Tanzania in 1952.^5,14^ Since then, there have been several outbreaks in Africa, Asia, Europe, and the Americas.^15^ CHIKV causes chikungunya fever. The acute stage of the disease typically spans 21 days^16^ and consists of the triad of fever, rash, and arthralgias. After the acute stage, the articular symptoms either resolve on their own or persist for weeks to years. Patients may develop chronic periarticular pain or arthritis that mimics rheumatoid arthritis or spondyloarthritis.^17^ It is estimated that approximately 52% of patients infected with CHIKV experience chronic articular pain.^5^ Although less common, neuropathic pain can also develop after CHIKV infection. A Brazilian large-scale prospective study reported that 22% of patients had myelitis after infection,^18^ which sometimes leads to central neuropathic pain at or below the level of the spinal cord lesion.^11^

Varicella zoster virus (VZV) is an alpha herpesvirus that infects children and causes chickenpox, which presents as pruritic, fluid-filled vesicles on the skin.^19^ After chickenpox resolves, VZV becomes latent in the dorsal root ganglia, cranial nerve ganglia, and autonomic ganglia.^11^ As cell-mediated immunity declines with age, VZV may reactivate and lead to the development of herpes zoster (commonly known as shingles), a rash with a dermatomal distribution.^20^ Approximately 10% of patients with herpes zoster experience postherpetic neuralgia (PHN) within a year of rash appearance.^21^ PHN is the most well-known example of post-viral neuropathic pain. It is typically described as a burning sensation and allodynia in the same dermatomal distribution as the rash.^11^

Human immunodeficiency virus (HIV) is the causative agent of acquired immunodeficiency syndrome.^22^ HIV is most frequently transmitted sexually but can also be transmitted through injection drug use, via blood transfusions, and vertically from mother to child.^23^ The virus progressively weakens the immune system by attacking CD4^+^ T cells, making patients more susceptible to opportunistic infections.^24^ HIV is also able to attack the peripheral and central nervous system (CNS), causing sensory polyneuropathies.^11,25^ Some patients with HIV report allodynia and burning distally in the lower limbs,^26^ and up to 50% of patients with HIV experience chronic pain in their lifetime.^11^

Poliomyelitis is caused by poliovirus and was one of the most acutely debilitating viral infections in the 20th century.^27^ The disease affected millions worldwide in the 1940s and 1950s, but due to widespread vaccination since the mid-1950s, polio is estimated to be 99% eradicated today, though it is still prevalent in some African, Asian, and South American countries.^11,27^ Permanent paralysis occurs in 1 of 200 infections,^11^ and 30% to 80% of patients experience post-polio syndrome (PPS).^28^ PPS presents with muscle weakness, fatigue, myalgia, arthralgia, neuropathic pain, and functional decline 10 to 15 years after poliovirus infection.^29^ Myalgia and arthralgia have been found to be the most common and bothersome symptoms of PPS.^30^

Severe acute respiratory syndrome (SARS) first emerged from Southeast Asia in 2003 and was caused by a novel SARS-associated coronavirus.^31^ Acutely ill patients presented with fever, nonproductive cough, myalgia, and dyspnea.^32^ After recovering from the infection, a proportion of patients experienced persisting symptoms, namely, musculoskeletal pain, fatigue, shortness of breath, and sleep issues.^31^ The 2020 COVID-19 pandemic is caused by a coronavirus (SARS-CoV-2), and experts hypothesized that COVID-19 could lead to a PVS similar to that experienced after SARS.^2^ Those predictions were correct; post-COVID syndrome, also known as “long COVID,” has been reported in a significant subset of resolved cases.^33^ In addition to chronic fatigue and insomnia, patients experienced muscle pain, joint pain, chest pain, and headaches.^34^ Neuropathic pain can also persist in the post-viral stage due to the virus’s effects on the peripheral and central nervous systems.^11^ COVID-19 infection increases risk for stroke and development of Guillain-Barre syndrome, which can lead to post-stroke pain^35^ and chronic neuropathic pain, respectively.^11^

Ebolavirus outbreaks have been occurring in central Africa since 1976,^4^ with the largest outbreak taking place from 2013 to 2016.^10^ Ebola virus disease has a mortality rate that ranges from 25% to 90% depending on the species.^36^ Despite the high mortality rate, there are many survivors (>17,000 from the 2013–2016 outbreak alone), a significant proportion of whom have experienced long-term symptoms. Myalgias, arthralgias, and headache are among the most frequent symptoms experienced by patients in the postinfectious stage.^4,10^ An observational cohort study found that out of 802 patients, 38% had long-term musculoskeletal pain and 35% had long-term headaches.^4^

Post-viral pain has also been reported in dengue^6,13^ and influenza B^3^ cases. There is a report on a rare case of longitudinally extensive transverse myelitis in a 15-year-old male following dengue fever.^6^ The patient presented with intense, debilitating pain in the lumbar region. Another case report on a patient with dengue discussed a 14-year-old female who presented with right-sided hip and buttock pain from sacroiliitis that began 10 days after developing dengue fever.^13^ Influenza B was linked to multifocal neuropathy and local myositis in a case report of a 47-year-old male who presented with aching and dysesthesia in the right arm and left leg weeks after influenza B infection.^3^

### Pathophysiology of Post-Viral Pain

The pathogenesis of post-viral pain is poorly understood, and laboratory studies investigating molecular pathways that cause the phenomenon are scarce. The causes of chronic pain after CHIKV^15^ and herpes zoster^37–39^ have been the most researched of the conditions discussed herein, although the mechanisms remain undefined in CHIKV. There are fewer studies on pathogenesis for other diseases, such as Ebola virus disease,^40^ COVID-19,^41–43^ and West Nile virus^44^; however, some researchers have postulated various theories.

Chronic articular pain is a common outcome in patients who have been infected with CHIKV, Ebola virus, and West Nile virus.^5,40,44^ Immune activation is a natural response to viral infections whereby the immune system is activated to help fight the virus. However, prolonged or excessive immune activation can lead to the release of inflammatory molecules, such as cytokines, which can sensitize nerves and contribute to the development of pain. The arthralgia experienced by patients is characterized by tissue destruction and the presence of inflammatory cytokines, such as interleukin (IL)-1β, IL-6, and tumor necrosis factor alpha.^15,44^ Infected fibroblasts produce interferon, which increases expression of prostaglandins in the cells. This pathway is thought to contribute to nociceptor activation and sensitization in arthritic joints. When this pro-inflammatory reaction continues without cessation, it may lead to the joint pain that persists long after recovery from the acute phase.^15^ Another cell type that can be infected by viruses are osteoclasts. Patients with CHIKV have been found to express high levels of RANKL/osteoprotegerin, which can indicate the presence of macrophage-derived osteoclasts, or cells that cause bone destruction.^15^ Tissue damage may also be caused by cross-reactive antibodies to host proteins.^40^

Post-viral pain can also be neuropathic in nature. The most common example of this is herpes zoster. When dormant VZV reactivates, it causes acute herpes zoster, also known as shingles.^20,37^ The virus then replicates and triggers an inflammatory immune response, which damages peripheral and central sensory neurons, leading to generalized necrosis in the skin, nerves, and ganglia.^38,39^ The damaged peripheral nerves may become unable to inhibit nociceptive signals and, as a result, the threshold for nociceptive pain is decreased and ectopic discharges are produced. This creates disproportionate pain response to nonpainful stimuli, a phenomenon called peripheral sensitization.^39^ Inflammation triggered by the active virus also causes central sensitization by impairing descending inhibitory pain pathways. The combination of damaged peripheral nerves and changes in the CNS is what causes the pain experienced in PHN.^39^ Neuropathic pain that persists post-COVID is also thought to be the result of virus-induced damage to the peripheral and central nervous systems.^11^

The pathophysiology of the pain related to immune activation and reactive oxygen species (ROS) in post-viral infections is complex and multifaceted. It involves a range of cellular and molecular mechanisms that can interact and amplify each other, leading to persistent pain and inflammation. Elevated ROS levels in the spinal cord can cause hyperexcitability in the nervous system and subsequent hyperalgesia without inflicting any nerve damage or tissue inflammation.^45^ In patients with chronic pain syndromes, production of ROS has been postulated to account for pain in the chronic phase, and inflammatory processes lead to pain in the acute phase. Therefore, the increased production of ROS in viral infections, such as influenza and COVID-19, may cause alterations in pain processing pathways and lead to chronic post-viral pain.^46,47^

**Figure 1.**
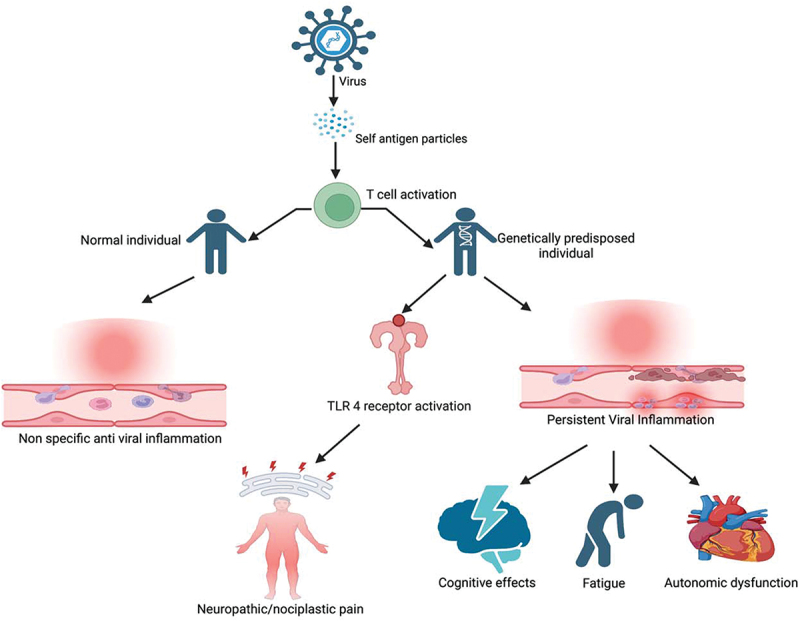
Simplified pathophysiology of post-viral syndromes.

More recently, it has been identified that viral antigens can present with a structure similar to self-antigen or cause release of self-antigens that trigger activation of autoimmune T cells. These autoimmune cells instead lead to the activation of Toll-like receptors (TLRs), which are involved in the innate immune response.^48^ TLRs are typically found in immune and glial cells of CNS. TLR activation can trigger neuroinflammation with the release of cytokines and chemokines, leading to altered nociception and inflammation in the absence of tissue damage or lesions in the somatosensory system.^49^ After infection control, neuroinflammation typically subsides in healthy individuals. However, in genetically vulnerable individuals, the inflammatory process continues to progress, resulting in persistent chronic pain. Moreover, it can lead to the disruption of neurotransmitter systems, including those involved in autonomic regulation resulting in autonomic dysfunction (Figure 1).^50^ This phenomenon is now recognized as nociplastic pain, which is different from nociceptive and neuropathic pain.^51^ Nociplastic pain is involved in a multitude of chronic widespread pain conditions like fibromyalgia and is likely implicated in post-viral pain.

### Diagnosis and Treatment of Post-Viral Pain

Existing literature on the diagnosis and treatment of post-viral pain after viral infections is scant. With the exception of PHN, which can be diagnosed when neuropathic pain persists beyond 3 months in the same distribution as the preceding dermatomal rash, there are no clear guidelines on the diagnosis of post-viral pain.^39^ The diagnosis is made when pain is preceded by a viral illness and cannot be explained by other conditions, such as rheumatoid arthritis, radiculopathy, and cancer.

Pain syndromes without a viral etiology are typically treated similarly to chronic pain.^11^ However, the French Infectious Diseases Society has published guidelines for the management of patients with CHIKV at various disease stages.^16^ In the postacute phase, analgesics and nonsteroidal anti-inflammatory drugs (NSAIDs) are used to relieve pain and reduce inflammation, with systemic corticosteroids such as prednisone reserved for exceptional cases. The choice of NSAID depends on the patient’s presentation and the clinician’s expertise as no specific class of NSAID has demonstrated superiority in treating post-CHIK symptoms.^16^ In addition, physiotherapy may also be used to improve range of motion, prevent muscle atrophy, and provide pain relief. In the chronic phase, the goal is to prevent further damage and enhance quality of life through the use of DMARDs (e.g., methotrexate).^16^ In an animal model, antiviral prophylactic drug research has yielded promising results but is yet to be replicated in human models.^52^

In the case of PHN and other neuropathic pains post-viral illness, the first-line treatments are typically alpha-2 delta ligands (e.g., pregabalin, gabapentin), tricyclic antidepressants (TCAs), or serotonin–norepinephrine reuptake inhibitors, with opioids and cannabinoids used as supplementary treatment if necessary.^39^ TCAs belong in one of two categories: secondary amines and tertiary amines. Tertiary amines, such as amitriptyline and clomipramine, have been shown to have greater efficacy in reducing PHN than secondary amines, such as nortriptyline and protriptyline.^39^ In cases of herpes zoster, antiviral medications and vaccination are effective in reducing the intensity of acute pain and preventing the development of PHN.^39^ However, there is no evidence to support the use of antiviral medications to prevent or treat post-viral pain and fatigue at this time.

There is also developing evidence for the efficacy of behavioral interventions in management of post-viral pain.^53^ A recent trial comparing pregabalin alone and pregabalin+cognitive behavioral therapy (CBT) for management of PHN showed improvement in neuropathic symptoms of burning and allodynia as well as pain-related catastrophizing in the CBT group. Moreover, those who completed CBT while taking pregabalin showed downregulation of mRNA expression of IL-6.^54^ Behavioral approaches, such as CBT or mindfulness-based interventions, are an important component of symptom management in post viral pain. In Sweden, intravenous immunoglobulin has been studied for post-polio pain, with 69% of patients reporting positive results.^55^ In a case study involving peripheral neuropathy caused by COVID-19, a combination of intravenous immunoglobulin (IVIG), steroids, gabapentinoids, antidepressants, tramadol, and topical agents were used for pain management, resulting in total pain relief.^56^ Though conventional analgesics and NSAIDs may provide temporary relief, the most effective treatments for neuropathic pain include gabapentinoids, antidepressants, tramadol, and topical agents.^11^

## Post-Viral Fatigue

### Viral Agents Associated with Post-Viral Fatigue

Fatigue is another common symptom that can persist after a viral infection. In some cases, the fatigue occurs after activity, whereas in other cases it can persist even at rest.^1^ There is also a difference between muscle weakness and brain fatigue that is associated with attention and cognitive deficits.^57^ Fatigue can impair patients’ ability to work or fulfill their roles in other areas and may drastically reduce quality of life. When the fatigue continues for more than 6 months, CFS may be diagnosed.^58^

In addition to causing post-viral pain, Ross River virus (RRV) has been found to be associated with post-viral fatigue.^58^ In a prospective cohort study, 13 out of 60 patients with confirmed RRV infection developed postinfectious fatigue syndrome.^1^ In the same study, 5 out of 68 patients with confirmed Epstein-Barr virus infection also developed postinfectious fatigue syndrome. With both infections, greater severity of acute illness was the strongest predictor of the severity of postinfectious fatigue.^1^

Fatigue is the most common symptom of PPS; over 90% of polio survivors have reported new-onset or increased fatigue, and 41% reported that fatigue interfered with their ability to be productive at work.^57^ More recently, COVID-19 has also been found to be associated with post-viral fatigue because a significant minority of patients have experienced continued fatigue and anhedonia after surviving the disease.^43,59^

### Pathophysiology of Post-Viral Fatigue

Much remains unknown about the pathophysiology of post-viral fatigue, though several studies have proposed mechanisms that may lead to the syndrome. Proposed mechanisms include persistence of virus in the body causing autoimmunity, immune dysfunction, and autonomic dysregulation.

RRV has been thought to cause post-viral fatigue by evading the immune system through antibody-dependent enhancement (ADE), inflammatory cytokine dysregulation, and mitochondrial disruption.^60^ ADE is a phenomenon in which preexisting antibodies facilitate viral entry into host cells rather than aiding antiviral immunity.^61^ This process leads to increased virulence. RRV-ADE was first observed in the 1990s.^60^ Once RRV enters macrophages, it disrupts cytokine expression, causing downregulation of pro-inflammatory cytokines, such as tumor necrosis factor, Interferon gamma inducible protein 10, while upregulating anti-inflammatory cytokines, such as IL-10. Dysregulation of the inflammatory cytokines has implications for the immune response to RRV and may undermine long-term immunity. The disequilibrium of cytokine expression also has potential to reduce the rate of ATP synthesis by mitochondria in lymphoblasts.^60^ The alteration in mitochondrial function may account for the long-term fatigue often experienced after RRV.

Recent studies have also suggested that immune activation by viral infections may contribute to abnormalities of the autonomic nervous system, contributing to post-viral fatigue syndrome. One potential mechanism is through the production of secondary autoantibodies, by molecular mimicry of the immune system, against the autonomic nervous system, leading to its dysregulation and a host of neurological symptoms.^62^ In addition, autoantibodies against α/β adrenoceptors and muscarinic receptors post-viral infection frequently lead to development of postural orthostatic tachycardia syndrome (POTS) and orthostatic intolerance (OH). Other mechanisms include cytokine storms.^63,64^

The chronic fatigue experienced as part of PPS has been thought to be due to poliovirus’s effect on the brain.^57^ Since the 1950s, histopathology reports have shown lesions caused by poliovirus in the hypothalamus, thalamus, caudate nucleus, putamen, and substantia nigra.^57^ Damage to these brain areas responsible for cortical activation may be responsible for the drowsiness and lethargy experienced in PPS. Magnetic resonance imaging studies of the brain have also found poliovirus lesions in the basal ganglia and reticular-activating system. Presence of hyperintense signal was correlated with fatigue severity and difficulty with attention and concentration.^57^ Lesions in the hypothalamus may impair the paraventricular nucleus’s ability to secrete corticotropin-releasing hormone, thereby reducing production of adrenocorticotropic hormone and hypothalamus-pituitary-adrenal (HPA) axis activity overall. The HPA axis plays an important role in cortical stimulation, and decreased HPA axis activity has been documented in patients with CFS.^57^ Thus, damage to the paraventricular nucleus and subsequent impairment of corticotropin-releasing hormone and adrenocorticotropic hormone production may be another mechanism through which poliovirus causes post-viral fatigue.^57^

### Diagnosis and Treatment of Post-Viral Fatigue

There are no agreed-upon diagnostic criteria for post-viral fatigue. The diagnosis is typically made when fatigue is preceded by a viral illness and cannot be explained by other conditions, such as anemia, adrenal insufficiency, and cancer.^65^ Similarly, there is no standardized protocol for the treatment of post-viral fatigue. Management depends on the individual patient’s illness experience and is typically focused on energy conservation.^66^ Lifestyle modifications, such as reducing daily activity and low-intensity aerobic graduated exercise, are commonly prescribed. CBT has also been suggested.^67^ Indeed, a recent trial of CBT for management of fatigue in post-COVID syndrome found a medium effect size improvement in fatigue over treatment as usual, which was maintained 6 months after treatment.^68^ Autonomic dysregulation (POTS and OH) is a major contributor of fatigue post-viral infection and should be evaluated in individuals experiencing fatigue in PVS. Treatment of POTS/OH with pharmacological (e.g., propranolol, ivabradine, midodrine) and nonpharmacological measures including graduated aerobic activity with mild resistance training can help alleviate fatigue symptoms.^69^ One case report from England demonstrated the successful use of electroacupuncture in a patient who experienced post-viral fatigue following an “influenza-like” illness,^70^ although larger-scale studies have not evaluated its efficacy and the case report was anecdotal.

## Post-Viral Sleep Disorders

### Viral Agents Associated with Post-Viral Sleep Disorders

In addition to pain and persistent fatigue, sleep disorders can develop after acute viral infection. Sleep abnormalities have been observed in SARS survivors who struggled with daily fatigue. When compared to healthy controls, patients post-SARS had more arousal disturbances and abnormal appearance of the electroencephalogram alpha frequency in approximately 50% of sleep on polysomnography.^31^ Sleep disorders have also been documented following infection with the newer coronavirus, COVID-19.^7^ After recovering from COVID-19, some previously healthy patients have developed sleep apnea, reducing the quality and restorative effects of their sleep.^71^ One small study found that out of 11 patients post-COVID with suspected sleep disorders, 4 showed rapid eye movement (REM) sleep without atonia (RWA) on video polysomnography.^72^ RWA is known to be a prodromal stage of REM sleep behavior disorder, which is characterized by abnormal body movement during REM sleep.^73^

Tick-borne encephalitis (TBE) is caused by TBE virus, which infects the CNS and typically causes inflammation in the spinal cord, brain stem, basal ganglia, thalamus, and cerebellum.^74^ One of the long-term sequelae of TBE is fatigue, and patients with TBE have been found to score their sleep-related quality of life lower than healthy controls did.^74^ Pneumonia-associated viral encephalitis was also linked to disordered sleep in the case report of a 70-year-old man who developed parkinsonism and lethargy. Polysomnography revealed a fragmented sleep–wake cycle and a fragmented non-REM–REM ultradian cycle, leading the authors to suggest that encephalitis may lead to sleep disorders and parkinsonism.^75^

### Pathophysiology of Post-Viral Sleep Disorders

Sleep disturbance is a common symptom experienced by adults living with HIV. Difficulty with sleep maintenance, short sleep duration, and disturbed sleep–wake cycle have been reported by patients with HIV.^76^ It has been suggested that these sleep issues may be the result of polymorphisms in the genes that regulate circadian rhythm. Genotyping showed that patients with HIV had polymorphisms in the *CLOCK, CRY1, PER1, PER2*, and *PER3* genes, which have been associated with sleep outcomes in prior research.^76^ The results indicated that longer HIV exposure may impact the effects that the polymorphisms have on circadian rhythm strength.^76^ It is possible that genetic modifications play a role in the development of sleep disorders in other viral illnesses as well.

Sleep disorders seen in patients post-SARS and post-COVID-19 have been believed to be caused in part by CNS infiltration and inflammation.^31,72^ Both coronaviruses have the potential to be neuroinvasive through an olfactory route. The discovery of RWA in a group of patients post-COVID-19 led researchers to hypothesize that the virus caused changes in the brain stem, which maintains atonia during physiologic REM sleep.^72^ This theory is supported by neuropathological studies that found that neuroinflammatory changes were most prominent in the brain stem.^72^

### Diagnosis and Treatment of Post-Viral Sleep Disorders

Similar to post-viral pain and post-viral fatigue, there are no specific diagnostic criteria for post-viral sleep disorders. There are also no treatment guidelines specific to post-viral sleep disorders, so patients are managed based on their presentation. For example, a patient with PVS experiencing sleep apnea would be treated much like a regular patient with sleep apnea. In a case series of patients post-COVID-19 who experienced new-onset sleep apnea, automated positive airway pressure therapy was successfully used to treat their condition.^71^ The authors suggested that sleep apnea should be part of the differential diagnosis in all cases of post-COVID-19 fatigue syndrome. Behavioral sleep management interventions, such as CBT for insomnia (CBT-I), and brief behavioral treatment for insomnia (BBTI) also hold promise for treatment of post-viral sleep disorders. These brief interventions focus on changing behaviors associated with poor sleep and, in the case of CBT-I, changing cognitions that interfere with sleep. Efficacy of both CBT-I and BBTI is well established. Moreover, these interventions are effective for management of disordered sleep for individuals with complex medical concerns, including chronic pain.^77,78^ More research is needed to reveal special considerations required when managing sleep issues in patients with various PVSs.

## Post-Viral Mental Health Sequelae

### Viral Agents Associated with Mental Health Sequelae

Development of significant mental health concerns following viral infection has been commonly noted in PVS. However, this area has received only limited attention prior to the recent SARS-CoV-2 pandemic.^79–81^ A recent meta-analysis examined rates of distress, depression, anxiety, and posttraumatic stress disorder (PTSD) following infection by SARS-CoV-1, H1N1, Middle East respiratory syndrome-CoV, H7N9, Ebola virus, or SARS-CoV-2. Rates of mental health problems following these infections were quite similar across virus populations and, pooled across mental health diagnoses and viruses, impacted 55% to 60% of individuals during acute illness and 11% to 22% of individuals after recovery from the virus. However, rates of specific mental health diagnoses were notably lower, with 5% to 10% of individuals reporting mild–moderate post-viral anxiety and/or depression, whereas 4% and 2% reported mild or moderate posttraumatic stress, respectively. There is no documented association between viral illness and severe mental illness.^82^

Notably, rates of mental health concerns among individuals with HIV are higher than those described above. Among people living with HIV (PLWH), rates of mood and anxiety disorders are two to six times higher than in the general population.^83^ In particular, rates of PTSD are much higher than has been reported in other post-viral populations, with some estimates placing the prevalence of PTSD among PLWH higher than 50%.^84^ It is important to note that the social context and chronic nature of HIV infection also differ dramatically from those of the other viral agents described above.

### Pathophysiology of Post-Viral Mental Health Concerns

The pathophysiology of post-viral mental health issues remains poorly understood. Putative mechanisms, physiological, socioenvironmental, or behavioral, are believed to be largely shared among viral illnesses. The immune response generated in response to a virus has been implicated as a causal generator of mental health problems.^85^ Numerous studies have demonstrated dysregulation of inflammatory markers in mental health disorders, and it is possible that virally induced changes in pro-inflammatory molecules cause mental health problems.^86–90^ Post-viral mental health problems may also be indirectly caused by other post-viral symptoms (i.e., pain, fatigue, sleep disruption) or by the impact of these symptoms on the ability to engage in meaningful behavior or social interaction. Indeed, social responses and stigmatization following infection with HIV or Ebola virus, such as loss of family relationships and friendships, can be extremely deleterious to mental health.^83,91^ Further research is needed to fully understand the multifactorial nature of this.

It is important to note that there is considerable concern among patients and providers regarding misattribution of causation for mental health problems following viral infection.^92,93^ Patients with PVS understandably stress biological causes for mental health concerns, given that symptoms are often met with invalidating responses from medical providers or other social relations. It is also possible that invalidation of other post-viral symptoms by providers, friends, or family contributes to distress.

### Treatment of Post-Viral Mental Health Concerns

Research on treatment of post-viral mental health problems is limited. To the best of the authors’ knowledge, no interventions have been developed or tested specifically for post-viral mental health problems, with the exception of interventions for PLWH. In the context of PLWH, a range of mental health interventions have been deemed effective, with particular support for CBT-based interventions and those delivered by a psychologist.^84,94^ It is likely that similar adapted interventions would be beneficial for PVS originating from other viral illnesses. Alternatively, there has been some promising evidence for the use of anti-inflammatory treatments for mental health disorders.^95^ Though this has not been investigated in the context of PVS, such approaches may hold potential as a mechanism-focused treatment.

## Limitations of this Review

This review has some limitations. The main limitations of this review are the unsystematic methods in finding, selecting, and appraising the previous studies. Though it thoroughly addresses typical viral infections, it does not discuss the less common viral variants that can potentially lead to chronic pain. This review offers a comprehensive overview of the proposed pathophysiological mechanisms underlying PVSs, and it is imperative to acknowledge that empirical evidence supporting these mechanisms is still evolving and not yet fully elucidated. Furthermore, there exists a notable lack of consensus regarding universally accepted definitive classification and standardized diagnostic criteria for post-viral disorders. This review does not analyze the effectiveness and limitations of proposed therapeutic approaches for post-viral disorders. The usefulness and limitations of potential therapy options for post-viral diseases are not examined in this review. Finally, this study did not examine the severe influence of PVSs on patients’ quality of life, everyday functioning, and socioeconomic well-being. Expanding the scope of this review would undoubtedly facilitate a more comprehensive understanding of the complexities surrounding PVSs.

## Future Considerations

The pathophysiology of post-viral pain syndromes is complex and remains unestablished. The COVID-19 pandemic demonstrated the prevalence of chronic pain in large population cohorts following viral infections.^8^ There is an urgent need for research on the viral pain mechanisms, including their interactions with the immune pathways that cause chronic pain. In addition, it is essential to identify the epigenetic risk factors that contribute to the development of chronic persistent pain in some but not all individuals following a viral infection. This will aid in the development of targeted therapeutic approaches for the delivery of specific drug molecules designed to interrupt chronic pain pathways following viral infection. It is also important to note that specific PVS symptoms, such as pain, fatigue, or sleep disturbance, do not occur in isolation. Not only do these symptoms interact with each other but they are, in and of themselves, multifaceted, biopsychosocial constructs. Future research needs to take steps to consider this complexity in context to fully understand post-viral pain.

## Conclusion

In conclusion, PVSs encompass persistent symptoms following viral infections, often including pain, fatigue, and sleep disturbances, which can significantly impact patients’ lives. Despite their prevalence, our understanding of the underlying mechanisms remains limited, with small-scale studies dominating the literature. Given the growing population of COVID-19 survivors facing long-term symptoms, urgent research is needed to unravel these mechanisms. It is worth noting that only a small fraction of patients achieve complete recovery from PVSs, emphasizing the need for targeted therapies. Enhanced pathophysiological insights may also lead to more efficient diagnosis and treatment, relieving health care system burdens. Ultimately, a deeper understanding of how viral illnesses interact with the CNS may pave the way for prevention strategies in the future.

## Financial Benefits to the Authors

The authors confirm they did not receive any financial benefits as a result of conducting this study.

## Previous Presentation of the Research/Manuscript

The authors confirm that this manuscript, in whole or in part, has not been previously presented or published.
